# Supplementary material for: Analysis of dynamic and widespread lncRNA and miRNA expression in fetal sheep skeletal muscle
Source: PeerJ. 2020 Sep 22;8:e9957. doi: 10.7717/peerj.9957 (PMC7518186; doi:10.7717/peerj.9957)
Supplement: Supplemental Information 17 [file peerj-08-9957-s017.pdf]

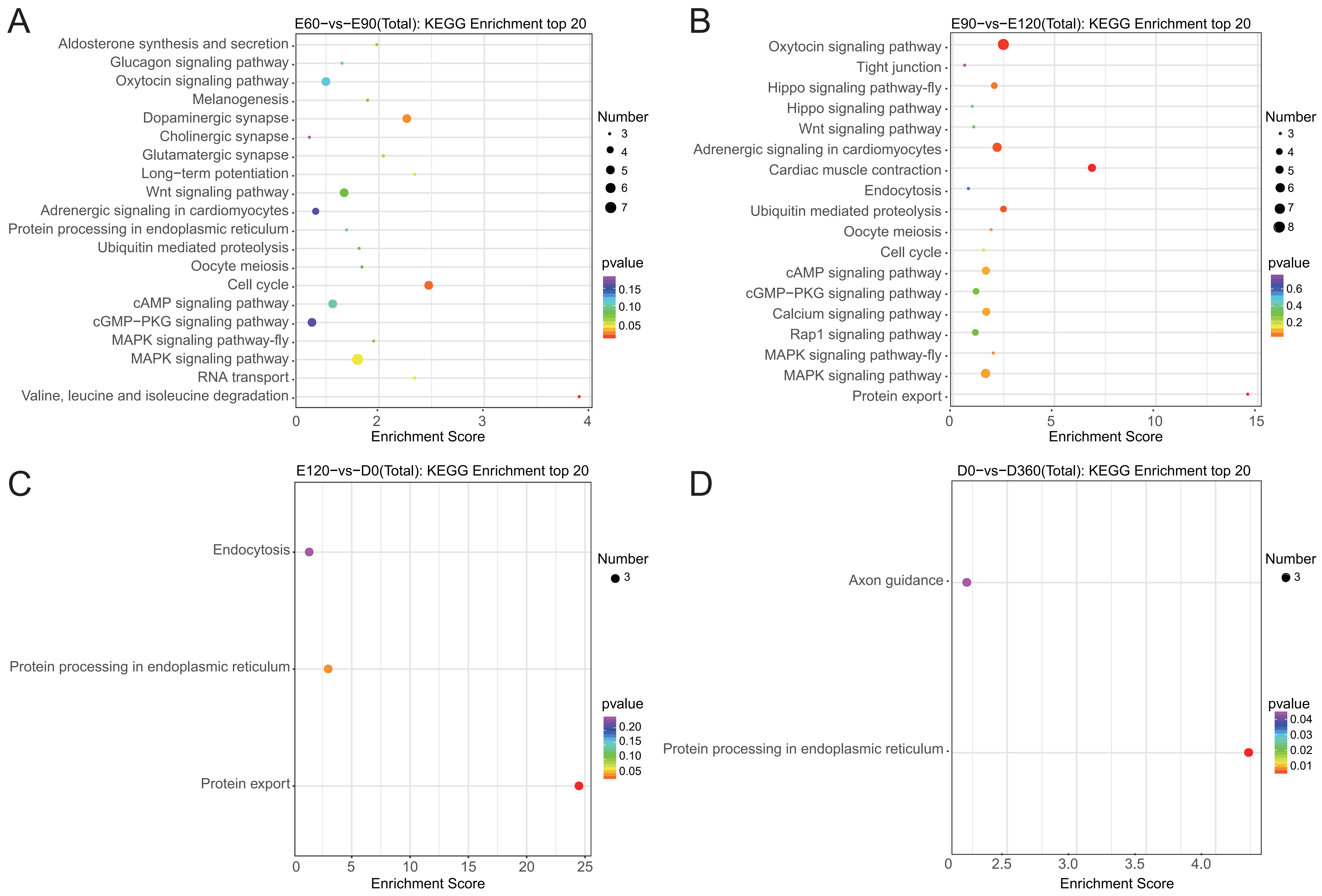
A
E60−vs−E90(Total): KEGG Enrichment top 20
Aldosterone synthesis and secretion
Glucagon signaling pathway
Oxytocin signaling pathway
Melanogenesis
Dopaminergic synapse
Cholinergic synapse
Glutamatergic synapse
Long−term potentiation
Wnt signaling pathway
Adrenergic signaling in cardiomyocytes
Protein processing in endoplasmic reticulum
Ubiquitin mediated proteolysis
Oocyte meiosis
Cell cycle
cAMP signaling pathway
cGMP−PKG signaling pathway
MAPK signaling pathway-fly
MAPK signaling pathway
RNA transport
Valine, leucine and isoleucine degradation
Number
3
4
5
6
7
pvalue
0.15
0.10
0.05
0
2
3
4
Enrichment Score
B
E90−vs−E120(Total): KEGG Enrichment top 20
Oxytocin signaling pathway
Tight junction
Hippo signaling pathway-fly
Hippo signaling pathway
Wnt signaling pathway
Adrenergic signaling in cardiomyocytes
Cardiac muscle contraction
Endocytosis
Ubiquitin mediated proteolysis
Oocyte meiosis
Cell cycle
cAMP signaling pathway
cGMP−PKG signaling pathway
Calcium signaling pathway
Rap1 signaling pathway
MAPK signaling pathway-fly
MAPK signaling pathway
Protein export
Number
3
4
5
6
7
8
pvalue
0.6
0.4
0.2
0
5
10
15
Enrichment Score
C
E120−vs−D0(Total): KEGG Enrichment top 20
Endocytosis
Protein processing in endoplasmic reticulum
Protein export
Number
3
pvalue
0.20
0.15
0.10
0.05
0
5
10
15
20
25
Enrichment Score
D
D0−vs−D360(Total): KEGG Enrichment top 20
Axon guidance
Protein processing in endoplasmic reticulum
Number
3
pvalue
0.04
0.03
0.02
0.01
0
2.5
3.0
3.5
4.0
Enrichment Score
