# Supplementary figures and images for: Analysis of dynamic and widespread lncRNA and miRNA expression in fetal sheep skeletal muscle
Source: PeerJ. 2020 Sep 22;8:e9957. doi: 10.7717/peerj.9957 (PMC7518186; doi:10.7717/peerj.9957)

E60-vs-E90(Total): Top 30 GO Term

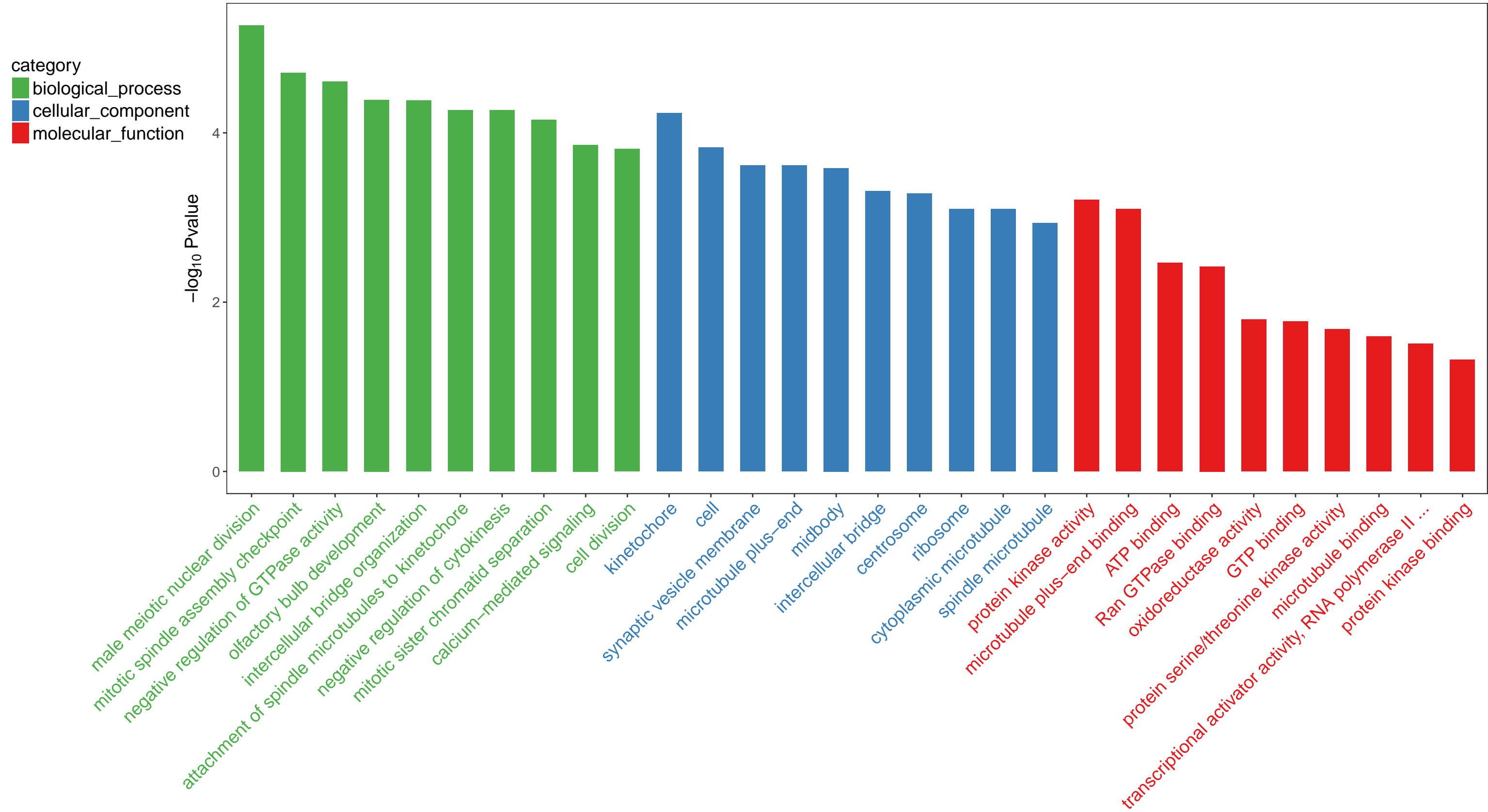

Supplement: Supplemental Information 18 [file peerj-08-9957-s018.pdf]

E90-vs-E120(Total): Top 30 GO Term

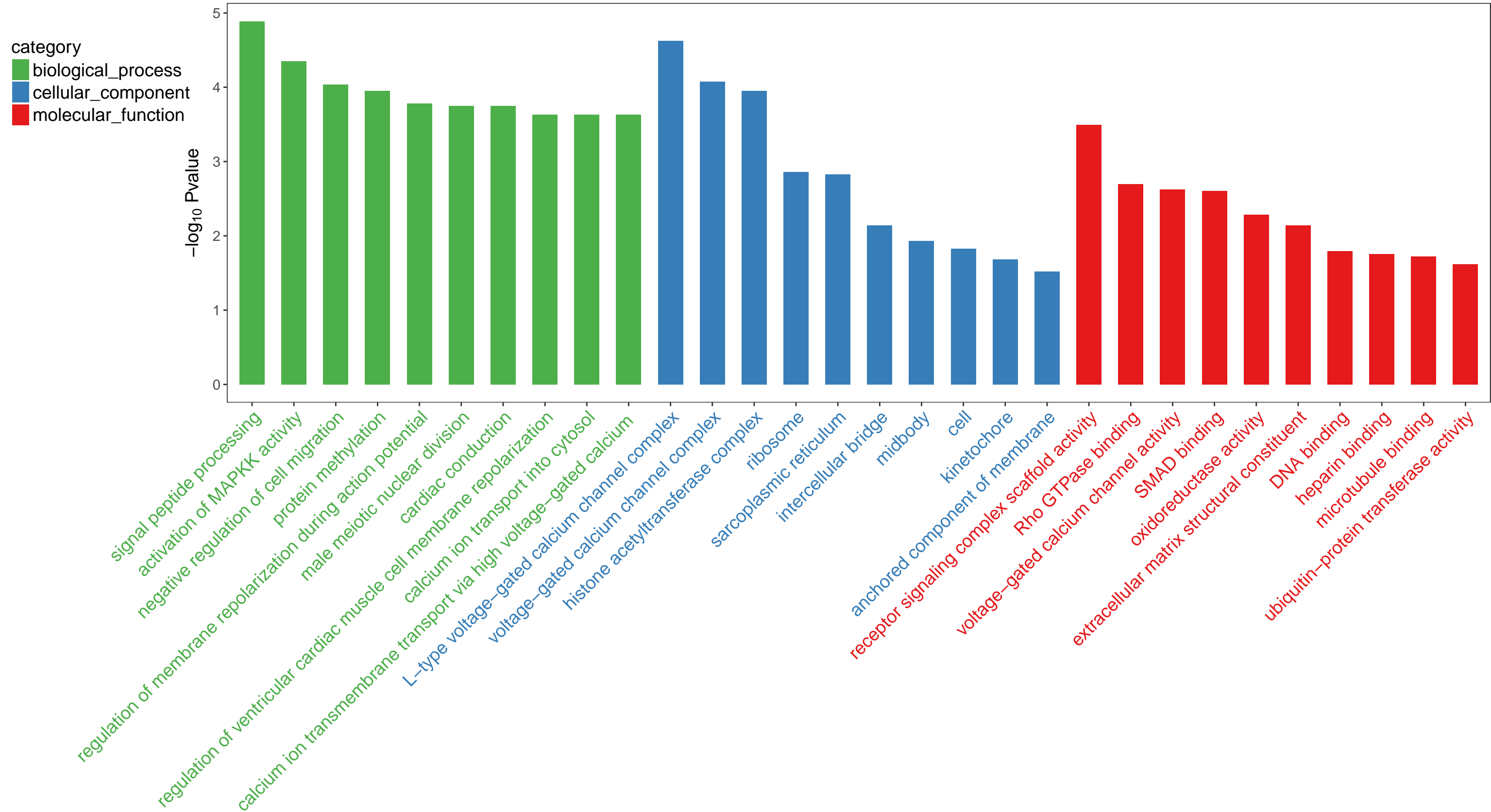

Supplement: Supplemental Information 19 [file peerj-08-9957-s019.pdf]

E120-vs-D0(Total): Top 30 GO Term

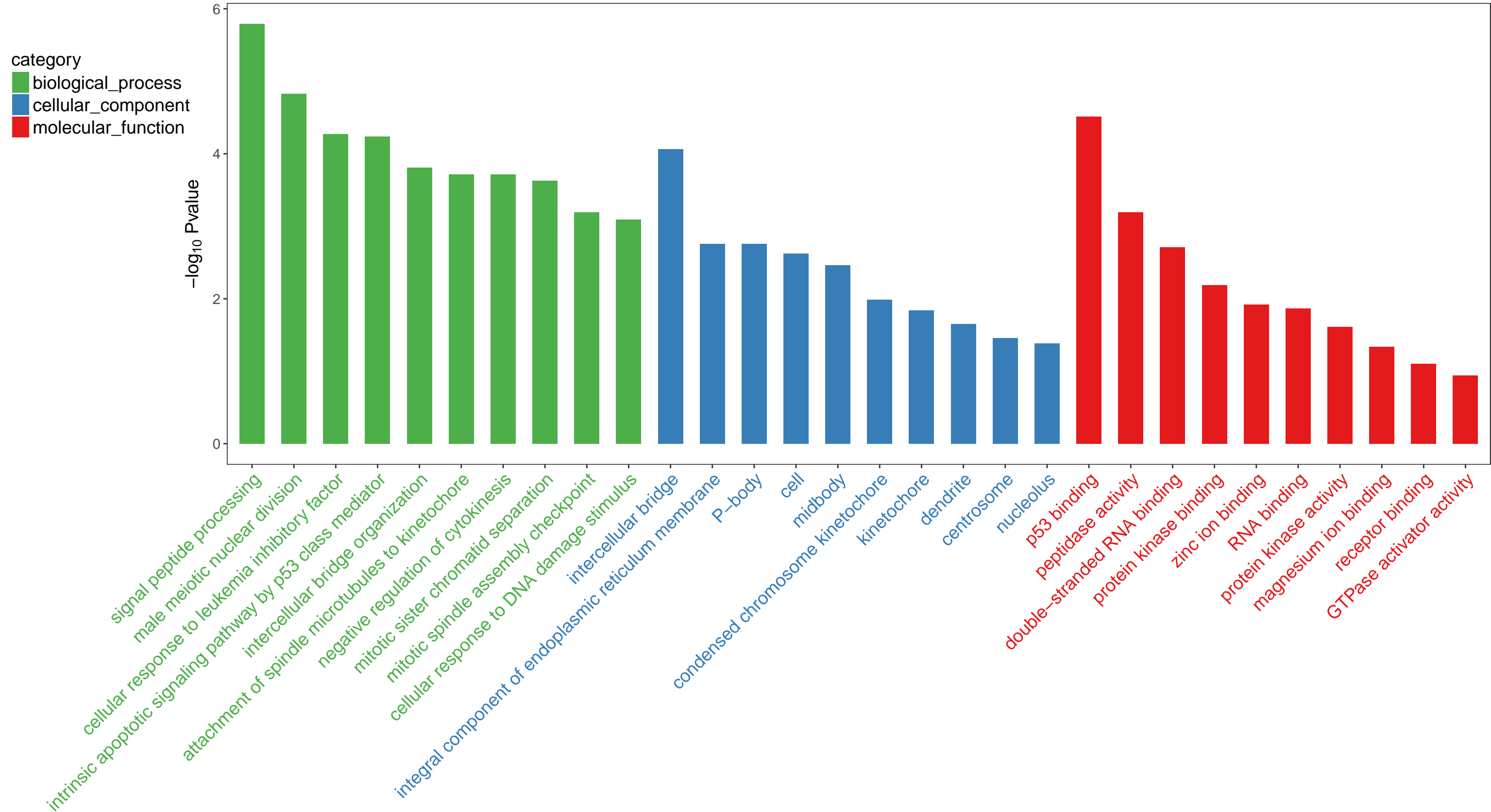

Supplement: Supplemental Information 20 [file peerj-08-9957-s020.pdf]

D0-vs-D360(Total): Top 30 GO Term

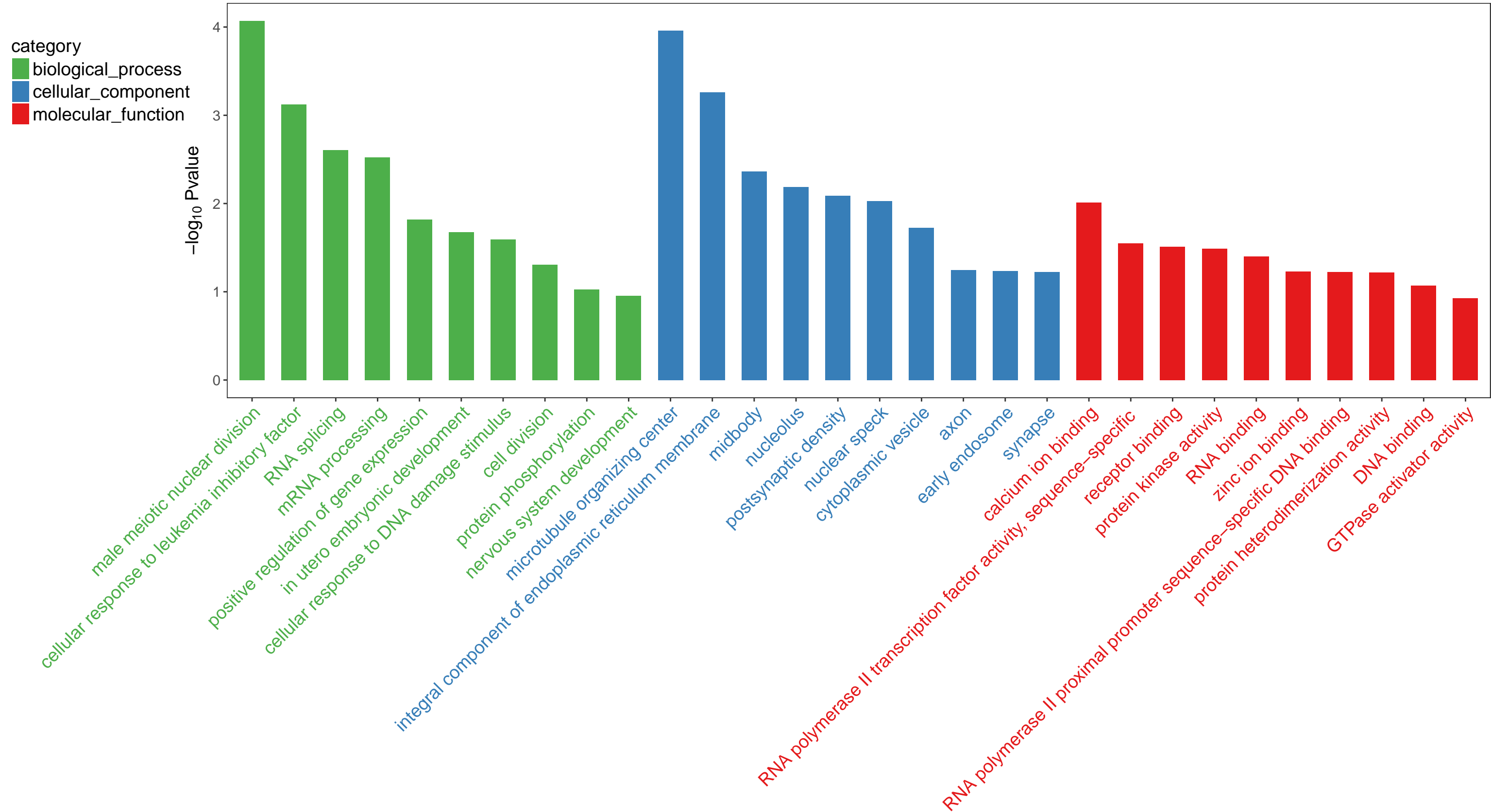

Supplement: Supplemental Information 21 [file peerj-08-9957-s021.pdf]

A

## Gene Ontology Classification (E60-vs-E90)

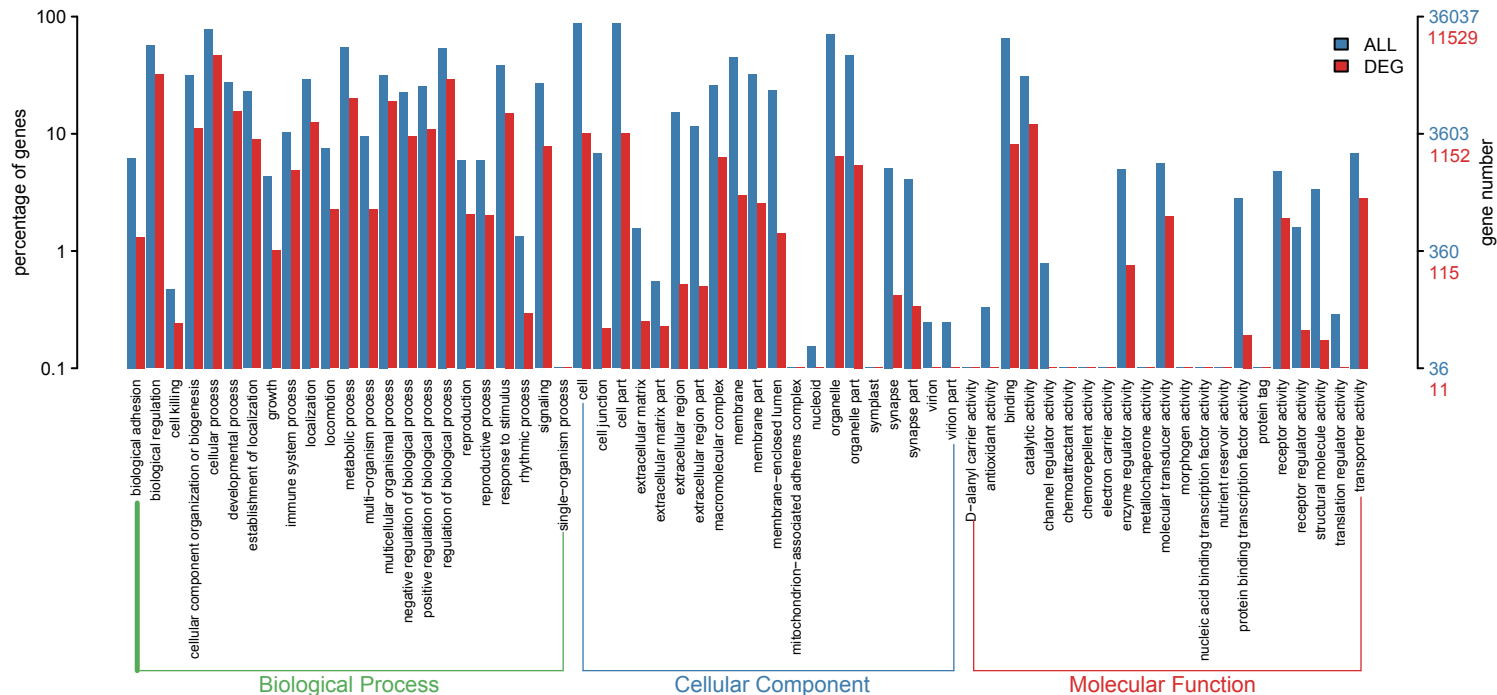

B

## Gene Ontology Classification (E90-vs-E120)

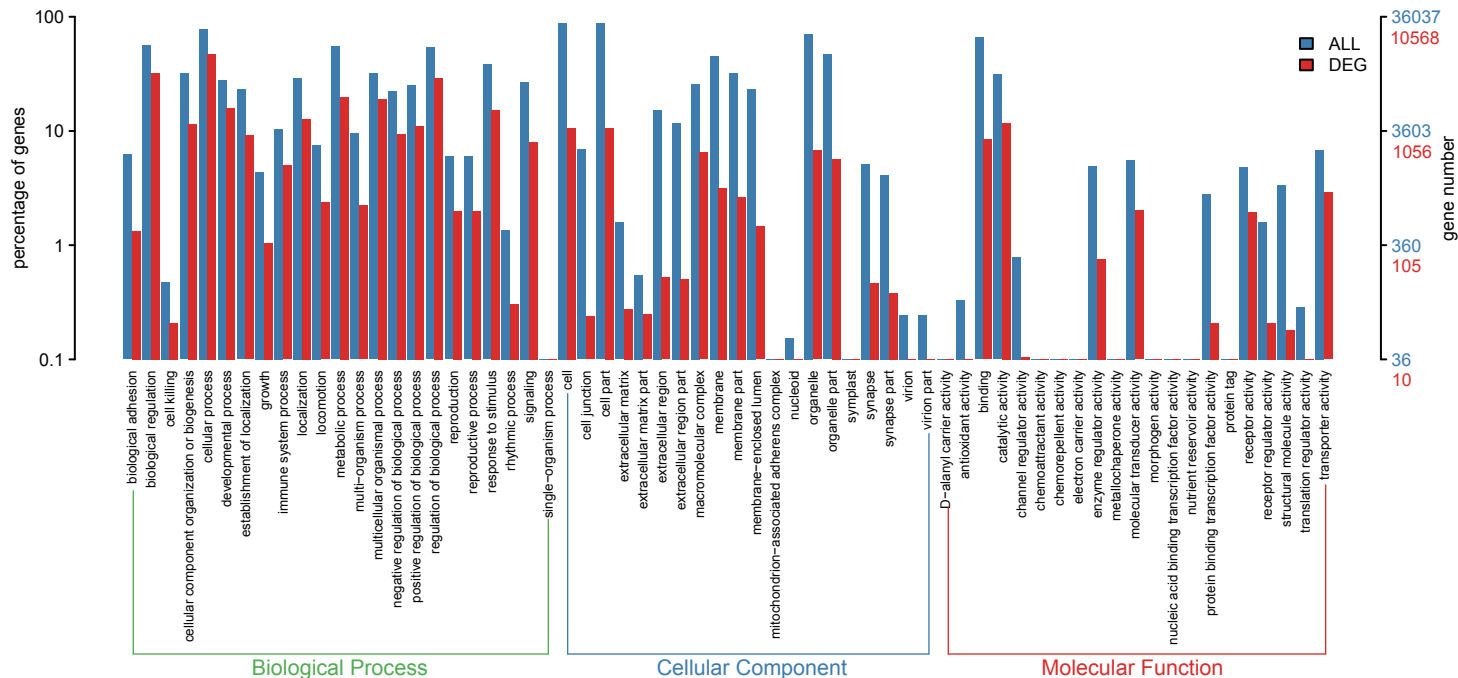

Supplement: Supplemental Information 22 [file peerj-08-9957-s022.pdf]

A

## Gene Ontology Classification (E120-vs-D0)

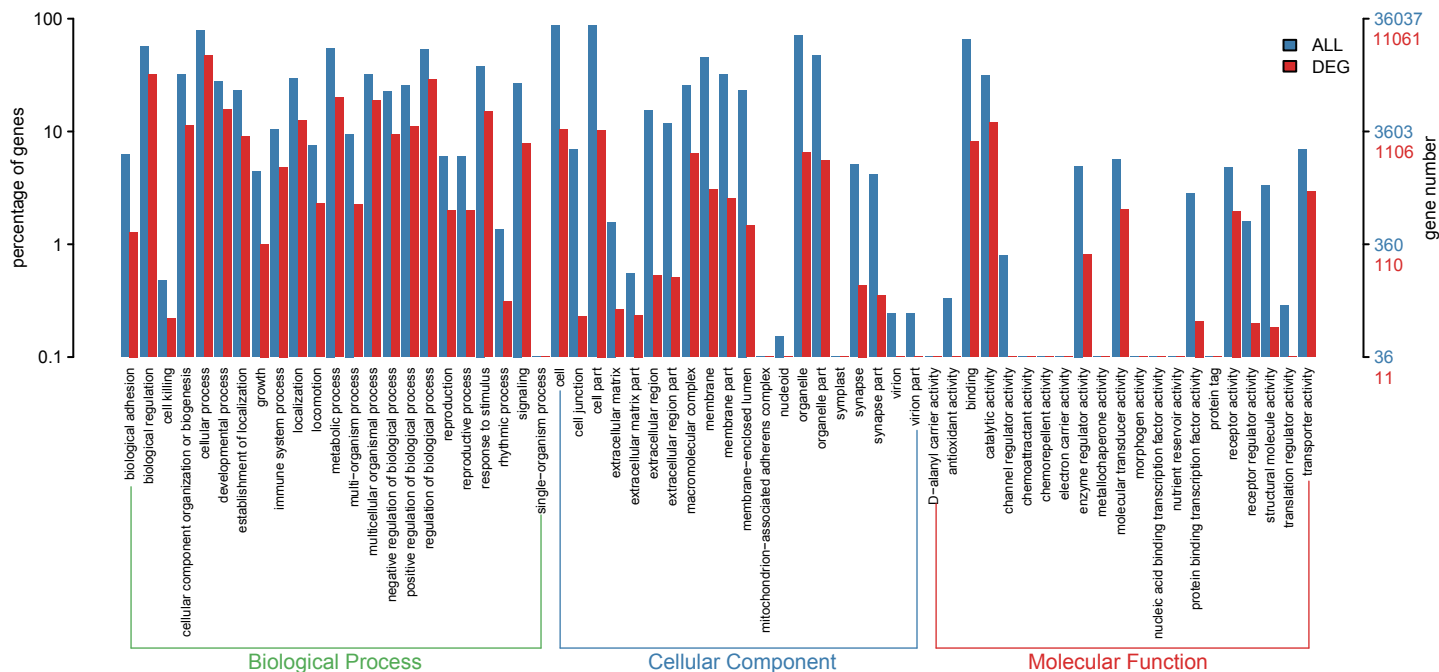

B

## Gene Ontology Classification (D0-vs-D360)

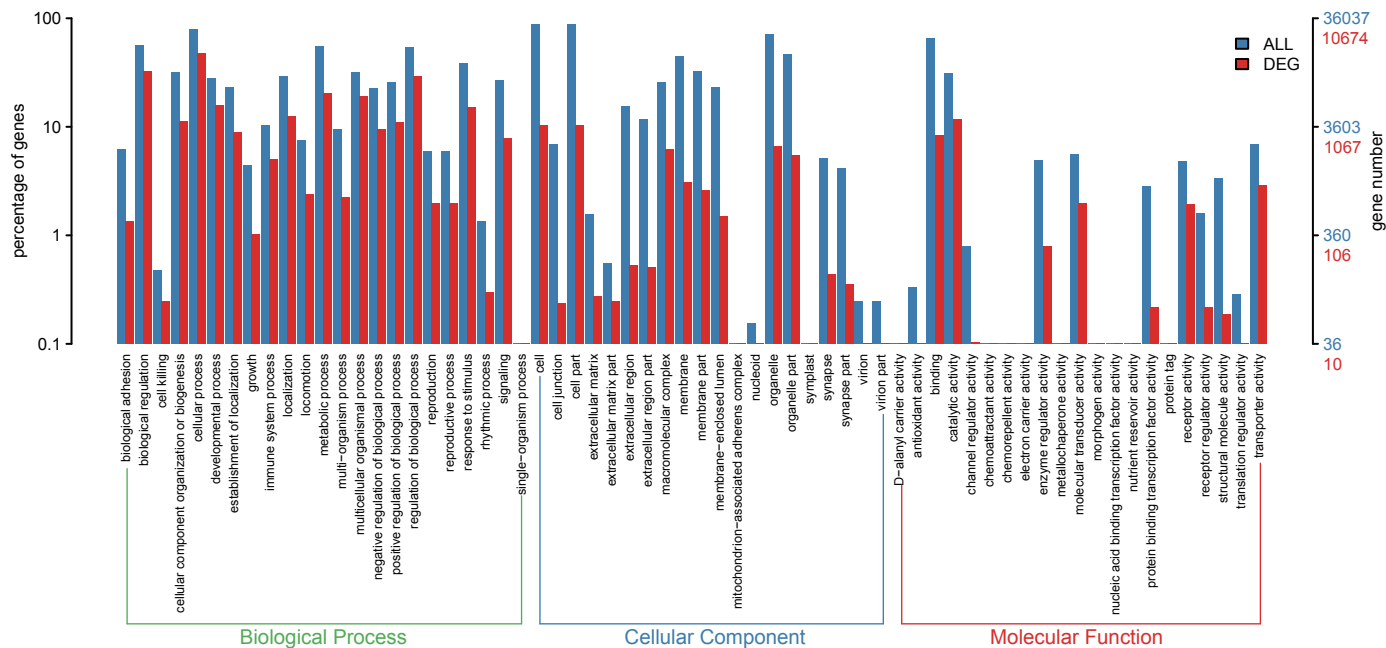

Supplement: Supplemental Information 23 [file peerj-08-9957-s023.pdf]
